# The Betacoronavirus PHEV Replicates and Disrupts the Respiratory Epithelia and Upregulates Key Pattern Recognition Receptor Genes and Downstream Mediators, Including IL-8 and IFN-λ

**DOI:** 10.1128/mSphere.00820-21

**Published:** 2021-12-22

**Authors:** Rahul K. Nelli, Juan Carlos Mora-Díaz, Luis G. Giménez-Lirola

**Affiliations:** a Veterinary Diagnostic Laboratory, Department of Veterinary Diagnostic & Production Animal Medicine, College of Veterinary Medicine, Iowa State University, Ames, Iowa, USA; University of Maryland School of Medicine

**Keywords:** ALI-REC, betacoronavirus, chemokines, cytokines, coronavirus, gene expression, IFN-λ1, IL-8, innate response, PHEV, RIG, TLR

## Abstract

The upper respiratory tract is the primary site of infection by porcine hemagglutinating encephalomyelitis virus (PHEV). In this study, primary porcine respiratory epithelial cells (PRECs) were cultured in an air-liquid interface (ALI) to differentiate into a pseudostratified columnar epithelium, proliferative basal cells, M cells, ciliated cells, and mucus-secreting goblet cells. ALI-PRECs recreates a cell culture environment morphologically and functionally more representative of the epithelial lining of the swine trachea than traditional culture systems. PHEV replicated actively in this environment, inducing cytopathic changes and progressive disruption of the mucociliary apparatus. The innate immunity against PHEV was comparatively evaluated in ALI-PREC cultures and tracheal tissue sections derived from the same cesarean-derived, colostrum-deprived (CDCD) neonatal donor pigs. Increased expression levels of TLR3 and/or TLR7, RIG1, and MyD88 genes were detected in response to infection, resulting in the transcriptional upregulation of IFN-λ1 in both ALI-PREC cultures and tracheal epithelia. IFN-λ1 triggered the upregulation of the transcription factor STAT1, which in turn induced the expression of the antiviral IFN-stimulated genes OAS1 and Mx1. No significant modulation of the major proinflammatory cytokines interleukin-1β (IL-1β), IL-6, and tumor necrosis factor alpha (TNF-α) was detected in response to PHEV infection. However, a significant upregulation of different chemokines was observed in ALI-PREC cultures (CCL2, CCL5, CXCL8, and CXCL10) and tracheal epithelium (CXCL8 and CXCL10). This study shed light on the molecular mechanisms driving the innate immune response to PHEV at the airway epithelium, underscoring the important role of respiratory epithelial cells in the maintenance of respiratory homeostasis and on the initiation, resolution, and outcome of the infectious process.

**IMPORTANCE** The neurotropic betacoronavirus porcine hemagglutinating encephalomyelitis virus (PHEV) primarily infects and replicates in the swine upper respiratory tract, causing vomiting and wasting disease and/or encephalomyelitis in suckling pigs. This study investigated the modulation of key early innate immune genes at the respiratory epithelia *in vivo,* on tracheal tissue sections from experimentally infected pigs, and *in vitro*, on air-liquid interface porcine respiratory cell cultures. The results from the study underscore the important role of respiratory epithelial cells in maintaining respiratory homeostasis and on the initiation, resolution, and outcome of the PHEV infectious process.

## INTRODUCTION

Porcine hemagglutinating encephalomyelitis virus (PHEV) is an enveloped, positive-sense, single-stranded RNA (ssRNA) virus that belongs to the order *Nidovirales*, family *Coronaviridae*, subfamily *Orthocoronavirinae*, genus *Betacoronavirus*, and subgenus *Embecovirus* (1). PHEV is related to murine hepatitis virus (MHV), bovine coronavirus (BCoV), and human coronavirus OC43 (HCoV-OC43) through a common ancestor (2). However, it is the only known *Betacoronavirus* (species *Betacoronavirus 1*) affecting pigs, with no evidence of infection in other species, including humans. PHEV is the causative agent of vomiting and wasting disease (VWD) and/or encephalomyelitis in suckling pigs (3–5). Recent *in vivo* infection studies from our group further demonstrated that PHEV-affected regions were characterized by a variable degree of lymphocyte and macrophage infiltration (6). However, the mechanisms causing this inflammatory infiltration are not completely understood.

Different experimental studies (6–10) and field outbreak investigations (4, 10, 11) described PHEV-associated respiratory signs early after infection. More recently, PHEV has been associated with respiratory disease in pigs (11). Actually, the virus was first isolated in 1970 from the nasal cavity of apparently healthy pigs during a routine diagnostic survey (8), and viral antigens were detected inside the cytoplasm of the epithelial cells lining nasal mucosa, bronchi, and bronchioles (12). Our group further demonstrated that, under *in vivo* and *ex vivo* experimental conditions, PHEV (67N strain) replicates in the upper respiratory tract epithelium and tonsil of cesarean-derived, colostrum-deprived (CDCD) neonatal pigs (6). Therefore, as for many other pathogens with respiratory tropism, respiratory epithelial cells could play a vital role in initiating and shaping the crucial defensive immunological processes during PHEV infections.

Besides the defensive role of the respiratory epithelium as a passive physical (cilia) and chemical barrier (mucins) (13, 14), it secretes a broad spectrum of molecules, including inflammatory and chemotactic mediators and antimicrobial substances (15, 16). As part of the upper respiratory tract, tracheal epithelia express a range of pattern recognition receptors (PRRs), including Toll-like receptors (TLRs), nucleotide-binding and oligomerization domain (NOD)-like receptors (NLRs), retinoic acid-inducible gene (RIG) I-like receptors (RLRs), membrane C-type lectin receptors (CLRs), and DNA receptors, which recognize and distinguish microbe-associated molecular patterns (MAMPs) from common flora and stimulate intracellular signaling pathways (17, 18). This signaling ultimately leads to the transcriptional upregulation of an antimicrobial and inflammatory response that can be time, location, and cell type dependent. Hence, it is particularly important to investigate PHEV infection and its immunopathogenesis in the upper respiratory tract, a primary site of PHEV infection.

In response to the need for alternative yet reliable approaches to traditional cell culture and animal infection models, organotypic airway cultures constitute an increasingly accepted paradigm shift for infection disease investigations across different animal species, including humans, cattle, sheep, mice, horses, cats, dogs, and pigs (19–27). In a previous study, we demonstrated that PHEV infects, replicates in, and induces cytopathic changes in porcine tracheal epithelial cells (PRECs) cultured in an air-liquid interface (ALI) (10). This model allows primary epithelial cells to differentiate into the pseudostratified epithelium resembling the epithelial lining of the tracheal region of the respiratory tract, recreating a physiological environment more representative of that *in vivo* than the traditional submerged culture systems. In addition, coculturing other immune cells in ALI cultures will lead to further evaluation of cell-cell and cell-virus interactions during infection. However, the mechanisms driving the modulation of the innate antiviral responses to PHEV are completely unknown. Therefore, the present study investigated key signaling mediators of the innate immune response against PHEV at the epithelial lining of the porcine respiratory tract, comparing both ALI-PREC cultures and tracheal tissue sections derived from CDCD neonatal pigs.

## RESULTS

### Microscopic relief contrast and ultrastructural analysis.

**FIG 1:**
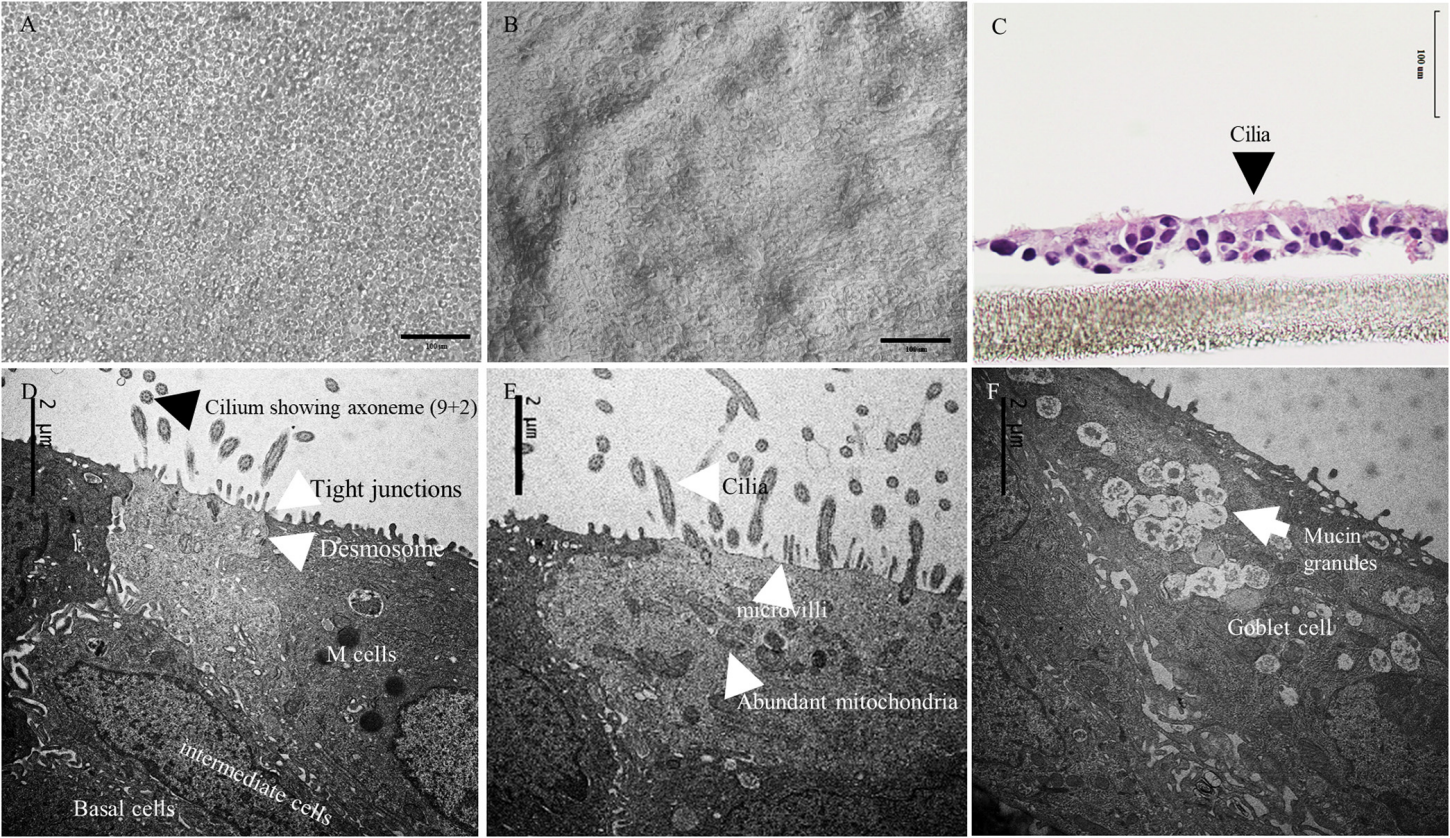
Morphology of air-liquid interface (ALI)-grown porcine respiratory epithelial cells (PRECs). (A and B) Relief contrast micrograph of nonadherent PRECs on transwell immediately after seeding (A) and on day 27 completely differentiated PRECs on transwell (B). (C) Paraffin-embedded H&E cross section of ALI-PRECs on transwell on day 27. (D) TEM images of day 27 ALI-PRECs, showing cilium axoneme, tight junctions, and desmosomes in a ciliated cell. Electron-dense secretory granules in some nonciliated cells, potential M cells; basal cells; intermediate cells. (E) Note the presence of mitochondria in ciliated cells, the powerhouse for ciliary movement. (F) Goblet cells were rich in mucin granules showing mucus. Representative images from three biological replicates.

Typically, pig tracheal sections yielded 300 to 500 million cells, and when seeded at a density of ∼20,000 cells/mm^2^ (Fig. 1A), approximately 25 to 30% confluence was achieved on day 1. By day 18, ALI-PRECs on transwells were completely confluent with no visible medium seepage; however, a shiny glaze that resembled mucus was observed on the surface of the cells growing on transwells under the microscope. Moreover, around the periphery of the transwell, some epithelial cells expressed cilia and started showing ciliary movement. The number of cilia continued to increase, and by day 27, cilium-expressing cells were at least 50% confluent (Fig. 1B and see Movie S1 in the supplemental material).

10.1128/mSphere.00820-21.2 MOVIE S1 Time course video showing mucociliary responses in mock-inoculated ALI-PRECs (6 hpi to 48 hpi). Download Movie S1, MPG file, 2.9 MB. Copyright © 2021 Nelli et al. 2021 Nelli et al. https://creativecommons.org/licenses/by/4.0/ This content is distributed under the terms of the Creative Commons Attribution 4.0 International license.

Paraffin-embedded cross sections prepared from 27-day-old ALI-PREC cultures on transwell inserts stained with hematoxylin and eosin (H&E) evidenced the presence of multilayered pseudostratified epithelial cells showing both ciliated and nonciliated regions (Fig. 1C). Transmitted electron microscopy (TEM) of these sections further confirmed the presence of ciliated cells (Fig. 1D) expressing cilium axoneme with abundant mitochondrion activity (Fig. 1E). The ultrastructural analysis also confirmed the presence of ciliated cells (Fig. 1E), mucin-secreting goblet cells (Fig. 1F), and other nonciliated cells such as brush cells, basal cells, and cells with electron-dense secretory granules, with the presence of tight junctions and desmosomes (Fig. 1D).

### Cellular characterization: immunocytochemistry and gene expression.

**FIG 2:**
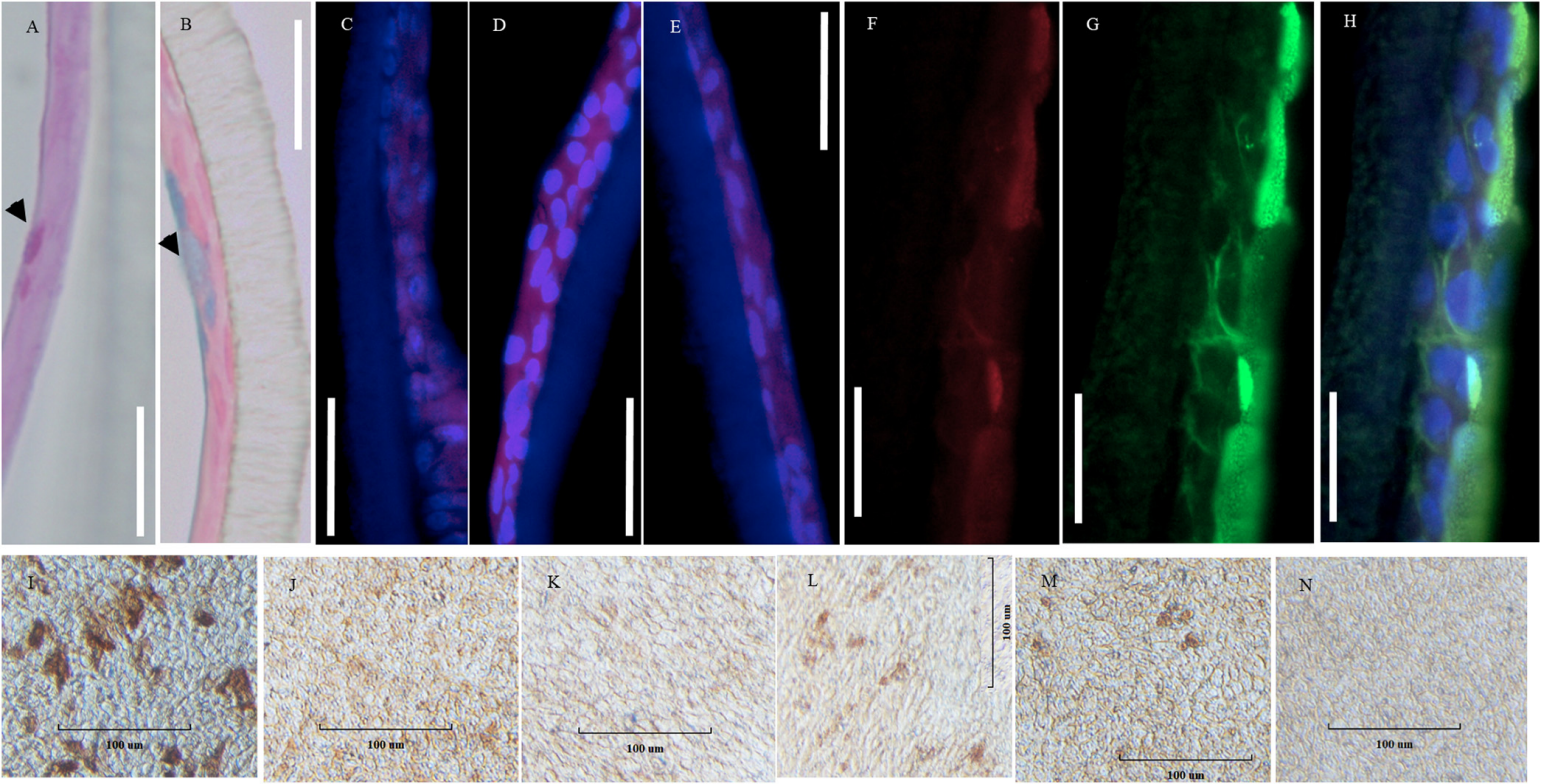
Cellular and receptor characterization of ALI-PRECs. (A and B) Paraffin-embedded cross sections of day 27 ALI-PRECs stained for periodic acid-Schiff stain (A), with arrowhead showing magenta red for neutral mucins, and alcian blue (B); note arrowhead indicating the blue stain for acidic mucins. (C to E) Immunofluorescence composite images of various cell markers (mouse monoclonal primary antibodies), where nucleus is stained blue (4′,6-diamidino-2-phenylindole [DAPI]) and red shows goat anti-mouse secondary antibody labeled with Alexa Fluor 647. (C) Tight junction marker occludin; (D) cell proliferation marker Ki67; (E) epithelial cell marker pancytokeratin. (F to H) Expression of sialic acids; biotinylated Maackia amurensis II (MAL II) was detected with streptavidin DyLight 650 conjugate, specific for α-2,3-linked sialic acids (F), while the expression of α-2,6-linked sialic acids was detected using fluorescein isothiocyanate (FITC)-labeled Sambucus nigra (SNA) lectin (G); (H) respective composite images showing predominant expression of α-2,6-linked sialic acids on ALI-PRECs. (I to N) Immunocytochemistry images of the surface of day 27 ALI-PRECs in transwell. Cells fixed in 4% paraformaldehyde were stained with ImmPRESS VR anti-mouse IgG horseradish peroxidase (HRP) polymer detection kit (MP-6402-15; Vector Laboratories) with mouse monoclonal antibodies. (I) Microfold cells; (J) ACE2; (K) CEACAM1/CD66a; (L) DPP4/CD26; (M) APN/CD13; (N) secondary antibody control. Brown represents a positive expression of the antibody; representative images from two biological replicates. Bar, 100 μm. Representative images from 3 biological replicates.

ALI-PRECs on transwells stained positive for periodic acid-Schiff (PAS) stain (Fig. 2A), which recognizes neutral mucins and alcian blue (Fig. 2B), which recognizes acidic sulfated mucosubstances, hyaluronic acid, or sialomucins. In addition, these cell cultures stained positive for tight junction marker occludin (Fig. 2C), cell proliferation marker Ki67 (Fig. 2D), epithelial cell marker pancytokeratin (Fig. 2E), and M-cell marker microfold antibody (Fig. 2I). However, the distribution of these markers was not uniform across the transwell. Gene expression studies in ALI-PRECs further confirmed the presence of proliferating cells (*PCNA*), goblet cells (*MUCIN2*), neuroendocrine cells (*CHGA*), stem cells (*LGR5*), brush cells (*SPIB isoform 1*), microfold cells (*GP2*), and basal cells (*KRT5*), similar to the porcine trachea (Table 1).

**TABLE 1:** qPCR determination of specific cell types (swine-specific genes) and receptors in ALI-PRECs and pig trachea^*a*^

Gene	NCBI accession no.	Specificity	ALI-PRECs	Trachea
PCNA	NM_001291925.1	Proliferating cells	+ (2/2)	+ (2/2)
MUCIN2	XM_021082584.1	Goblet cells	+ (2/2)	+ (2/2)
CHGA	NM_001164005.2	Neuroendocrine cells	+ (2/2)	+ (2/2)
LGR5	XM_021090898.1	Stem cells	+ (2/2)	+ (2/2)
SPIB isoform 1	XM_003127365.3	Brush cells	+ (2/2)	+ (2/2)
GP2	XM_003124571.4	Microfold cells	+ (2/2)	+ (2/2)
KRT5	XM_003126173.4	Basal cells	+ (2/2)	+ (2/2)
DPP4/CD26	NM_214257.1	Coronavirus receptors	+ (2/2)	+ (2/2)
APN/ANPEP	NM_214277.1	Coronavirus receptors	+ (2/2)	+ (2/2)
ACE2	NM_001123070.1	Coronavirus receptors	+ (2/2)	+ (2/2)
CEACAM1/CD66a	XM_021094420.1	Coronavirus receptors	+ (2/2)	+ (2/2)

a The presence and absence of the receptors are represented by + and −, respectively. Values in parentheses were the number of biological samples tested positive per total tested.

### Viral receptor distribution.

ALI-PRECs were stained for common viral protein coronavirus receptors, including sialic acids, ACE2, CEACAM1, DPP4, and APN. The expression of sialic acids on ALI-PRECs was evidenced by the relative abundance of Sambucus nigra lectin (SNA) and Maackia amurensis II lectin (MAL II), specific for α-2,6-linked sialic acids and α-2,3-linked sialic acids, respectively. However, the expression of α-2,6-linked sialic acids (Fig. 2G and H) was dominant compared to α-2,3-linked sialic acid receptors in ALI-PRECs (Fig. 2F and H). Although ACE2 (Fig. 2J), CEACAM1 (Fig. 2K), DPP4 (Fig. 2L), and APN (Fig. 2M) receptors were diffusely expressed (stained) on the surface of ALI-PRECs, further transcriptional analysis confirmed the presence of ACE2, CEACAM1, DPP4, and APN in ALI-PRECs as in their natural counterpart, i.e., tracheal epithelia *in vivo* (Table 1).

### PHEV replicates and disrupts ALI-PREC cultures.

**FIG 3:**
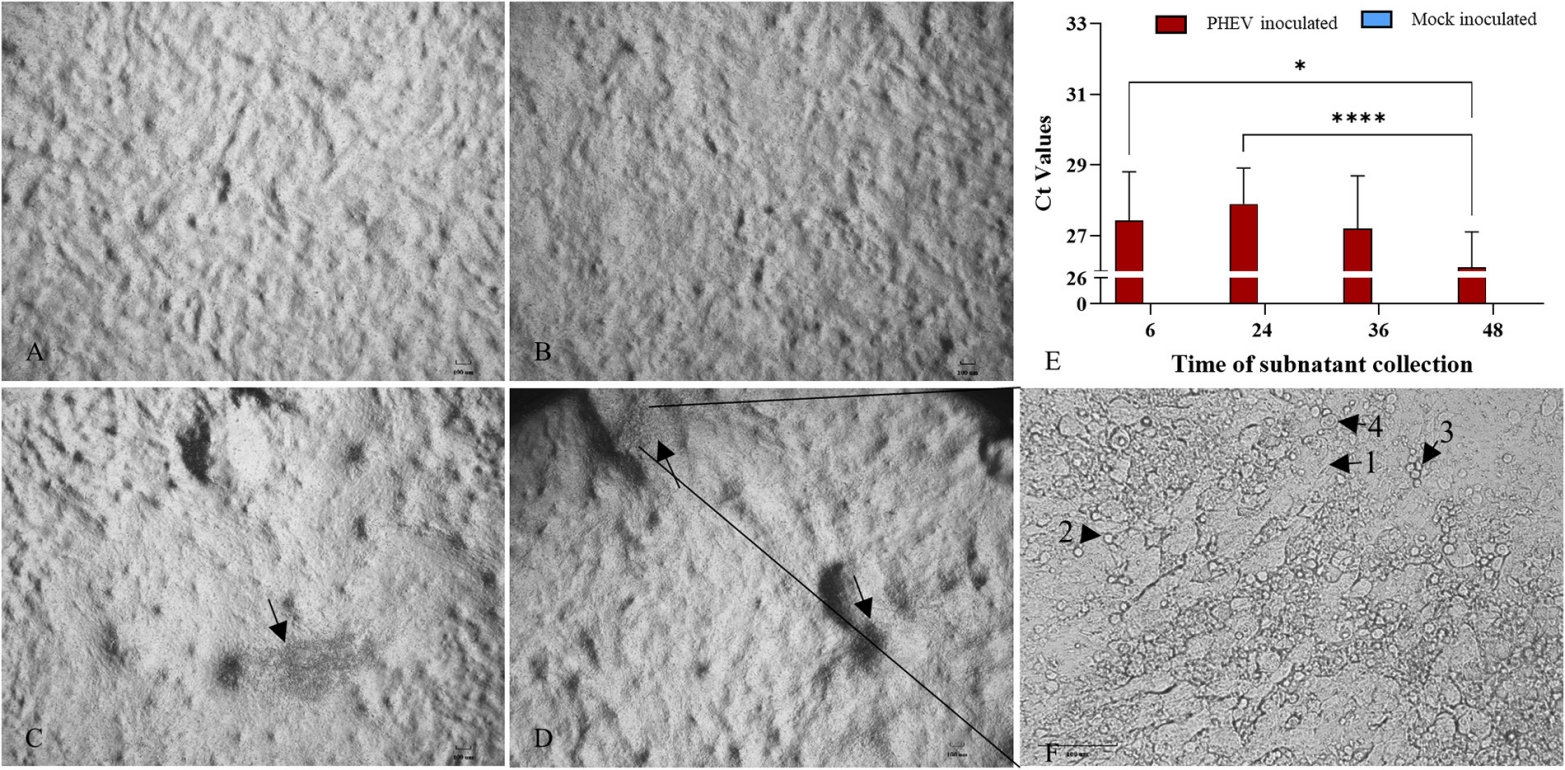
ALI-PREC susceptibility toward PHEV infection. (A to D) Completely differentiated ALI-PRECs (day 30) treated with infection medium only, i.e., without virus (mock inoculated) (A), and (B) with HA (titer of 128) of PHEV 67N for 24 hpi, (C) 36 hpi (arrow, area of CPE), and (D) 48 hpi (arrows, areas of CPE). (F) Magnification (×200) of CPE areas at 48 hpi (note the cytopathic effects on ALI-PRECs: [1] cytoplasmic stranding, [2] rounding of cells, [3] cluster of rounded cells, and [4] vacuolation of cells). Bar, 100 μm. Representative images from two biological and three technical replicates. (E) Detection of PHEV nucleocapsid gene using reverse transcription-qPCR. RNA from the subnatants collected from ALI-PRECs treated with PHEV was analyzed using RT-qPCR developed by ISU and Tetracore. Collection time is shown in hours. A sample volume of 5 μl of extracted sample RNA along with internal control was added to the qPCR master mix. All qPCRs were performed with a negative extraction control (NEC), a positive extraction control (PEC), and a no-template control (NTC) included in each run. Samples from two biological replicates and three technical replicates. Statistical analysis was performed using Fisher’s LSD multiple-comparison test (GraphPad Prism 9.0.1). *, *P* value < 0.01, and ****, *P* value < 0.0001.

ALI-PRECs appeared normal and showed active ciliary movement for up to 24 h postinoculation (hpi) in both mock- (Fig. 3A) and PHEV-inoculated (Fig. 3B) cultures. The virus-inoculated cells started showing cytopathic changes around 36 hpi (Fig. 3C) and increased over 48 hpi (Fig. 3D) compared to mock-inoculated cells (Movies S1 and S2). Cytopathic effects (CPE) included ciliary destruction (Movie S2), cytoplasmic stranding, vacuolation, rounding of cells, clusters of rounded cells, cell shrinkage, and detachment of cells exposing the transwell membrane of PHEV-inoculated ALI-PRECs (Fig. 3F). PHEV-affected areas presented a “leathery or crusty” appearance compared to unaffected areas in ALI-PRECs (Fig. 3C and D). Once the integrity of ALI-PRECs was breached, the virus started to shed into the bottom subnatants of the plate well medium between 6 and 48 hpi, as evidenced by the RT-qPCR results (Fig. 3E).

10.1128/mSphere.00820-21.3 MOVIE S2 Time course video showing mucociliary responses in PHEV-inoculated ALI-PRECs (6 hpi to 48 hpi). Download Movie S2, MPG file, 3.1 MB. Copyright © 2021 Nelli et al. 2021 Nelli et al. https://creativecommons.org/licenses/by/4.0/ This content is distributed under the terms of the Creative Commons Attribution 4.0 International license.

### Expression profile of innate immune genes in PHEV-infected ALI-PRECs.

**FIG 4:**
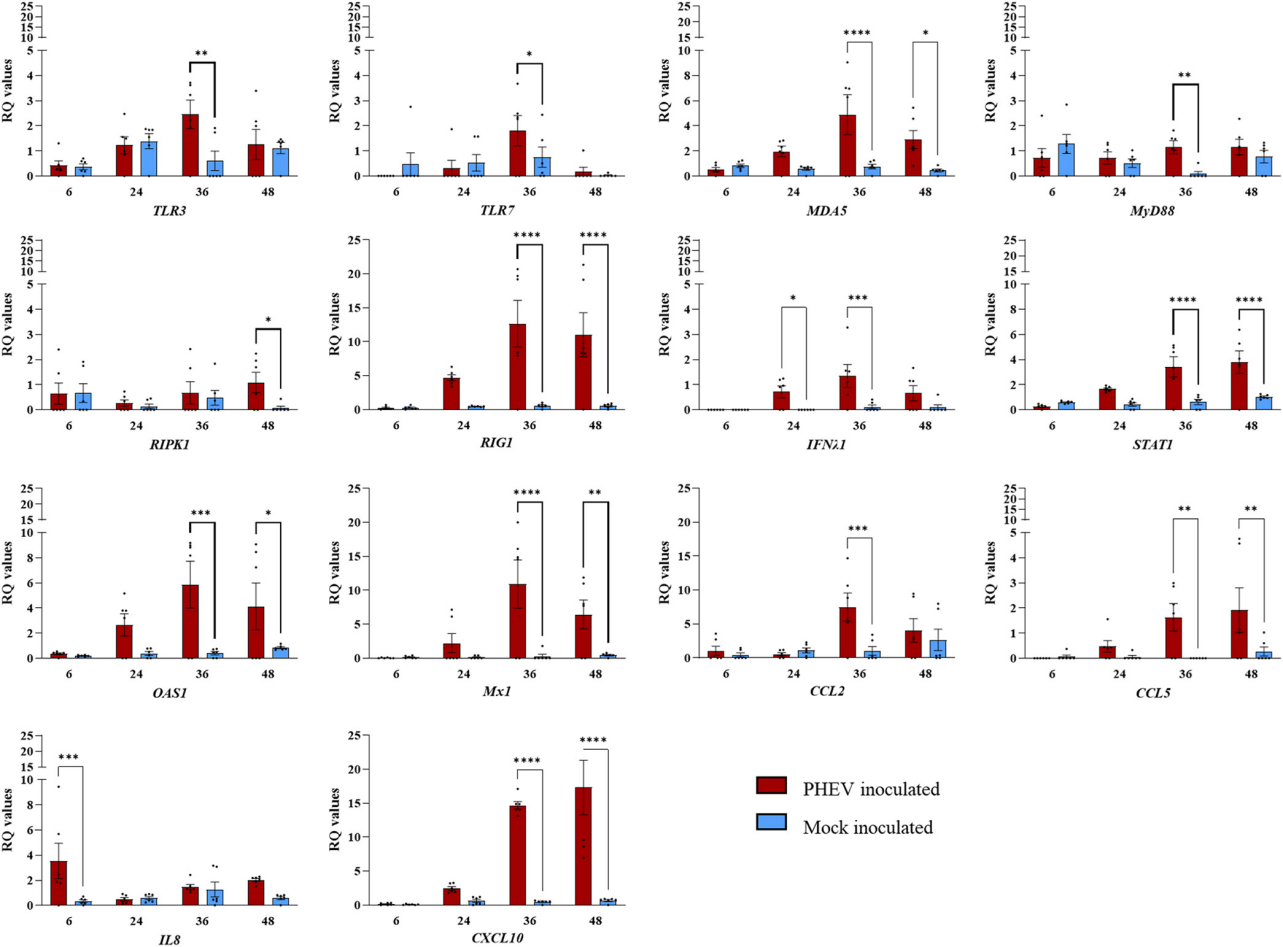
Transcriptional regulation of early innate immune genes in ALI-PRECs inoculated with PHEV or mock inoculated with culture medium. Bar graph showing relative quantification (RQ) levels of 14 porcine genes (*TLR3*, *TRL7*, *MDA5*, *MyD88*, *RIPK1*, *RIG1*, *IFNλ1*, *STAT1*, *OAS1*, *Mx1*, *CCL2*, *CCL5*, *IL8*, and *CXCL10*) measured in ALI-PRECs treated with HA (titer of 128) of PHEV and mock inoculum at respective hours postinfection (*x* axes). RQ values were calculated using the 2^−ΔΔ^*^CT^* method. The data are normalized against the geometric mean for three endogenous control genes (*EIF3K*, *PPIA*, and *RPL10*). This graph is generated from three technical replicates and two biological replicates (2 pigs). Statistical analysis was performed using Fisher’s LSD multiple-comparison test (GraphPad Prism 9.0.1). *, *P* value < 0.05; **, *P* value < 0.001; ***, *P* value < 0.005; ****, *P* value < 0.0001.

Following transcriptional analysis of 45 target genes, a significant increase in the expression of PRR *TLR3*, melanoma differentiation-associated protein 5 (*MDA5*), and *RIG1* genes was observed in PHEV-inoculated ALI-PREC cultures at 36 hpi (Fig. 4). Even though there was an increased expression of intracellular PRRs such as *NLRP3* and *NOD2*, their expression was variable and not statistically significant (data not shown). The expression levels of the myeloid differentiation primary response 88 (*MyD88*) gene, a key mediator of the inflammatory pathway, were punctually upregulated at 36 hpi in ALI-PRECs, while the expression levels of the cell death mediator receptor-interacting serine/threonine-protein kinase 1 (*RIPK1*) gene were significantly upregulated by 48 hpi (Fig. 4). Interestingly, there were no significant differences in the expression levels of both type 1 (*IFNα1* and *IFNβ1*) and type 2 (*IFNγ*) interferon genes, but the expression levels of the type 3 (*IFNλ1*) interferon gene were significantly upregulated at 24 hpi in ALI-PRECs (Fig. 4). The interferon stimulatory genes myxovirus resistance gene 1 (*Mx1*) and 2-5-oligoadenylate synthetase 1 (*OAS1*) were upregulated considerably by 36 hpi. Out of the four Jak-STAT signaling molecules (suppressor of cytokine signaling 1 [*SOCS1*], *SOCS3*, *STAT1*, and *STAT4*) analyzed in this study, only the *STAT1* gene was significantly upregulated (36 to 48 hpi) in ALI-PRECs following infection with PHEV. Consistent with this pattern, chemokine *CCL2*, *CCL5*, and *CXCL10* genes were also upregulated at 36 and 48 hpi, while the expression of *CXCL8/IL8* genes was >2-fold significantly higher at 6 hpi than at the subsequent time points evaluated (Fig. 4). Interestingly, the three proinflammatory cytokine genes *IL1β*, *IL6*, and *TNFα* were poorly expressed through the course of the study, with no significant differences between PHEV- and mock-inoculated ALI-PRECs (data not shown). The expression levels of TIR-domain-containing adapter-inducing interferon beta (*TRIF*) and TNF receptor-associated factor (*TRAF*) genes were not significant throughout the study. Moreover, although there was differential regulation of other innate immune response-related genes such as *MHC1*, *MHC2*, *NF p105*, *NF p50*, *NFkB2*, interferon regulatory factor 3 (*IRF3*) and *IRF7*, *AP1*, and *EGFR* in PHEV- versus mock-inoculated ALI-PRECs, the expression levels were variable across replicates and, therefore, not statistically significant (data not presented).

### Expression profile of innate immune genes in tracheal tissues from PHEV-infected pigs.

In a previous study (6), we reported the detection of significant PHEV RNA levels in tracheal tissues collected from pigs experimentally inoculated with PHEV and necropsied on day postinfection (dpi) 5. As a continuation, in the present study, we performed transcriptome analysis (45 target genes) of these tracheal tissues in comparison to their corresponding mock-inoculated pigs as the negative control. Note that PREC cultures derived from trachea tissue sections of the mock-inoculated control pigs.

**FIG 5:**
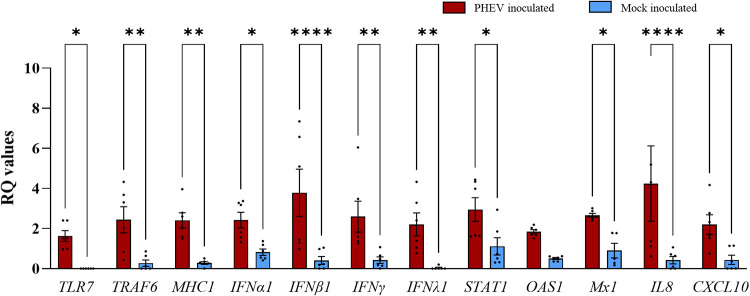
Transcriptional regulation of early innate immune genes in porcine tracheal tissues collected from pigs experimentally inoculated with PHEV or mock inoculated with culture medium. Bar graph showing relative quantification (RQ) levels of 12 porcine genes (*CXCL10*, *IFNα1*, *IFNβ1*, *IFNγ*, *IFNλ1*, *IL8*, *MHC1*, *Mx1*, *OAS1*, *STAT1*, *TRL7*, and *TRAF6*) measured in 5-day-postinfection snap-frozen pig trachea treated with HA (titer of 128) from PHEV and mock inoculum. RQ values were calculated using the 2^−ΔΔ^*^CT^* method. The data are normalized against the geometric mean for three endogenous control genes (*EIF3K*, *PPIA*, and *RPL10*). This graph is generated from three technical replicates and two biological replicates (2 pigs). An amount of 20 ng of RNA (cDNA) is used in each SYBR-green PCR. Statistical analysis was performed using Fisher’s LSD multiple-comparison test (GraphPad Prism 9.0.1). *, *P* value < 0.05; **, *P* value < 0.005; and ****, *P* value < 0.0001.

In response to PHEV infection, there was more than a 1-fold increase in the expression of *TLR7*, *TRAF6*, *MHC1*, *IL8*, *CXCL10*, *IFNα1*, *IFNβ1*, *IFN*γ, *IFNλ1*, *Mx1*, and *STAT1* genes, with a *P* value of <0.05 (Fig. 5). Even in the tracheal tissue, all proinflammatory cytokine genes (*IL1β*, *IL6*, and *TNFα*) along with other innate immune genes were either poorly expressed or variable between two pigs, with no significant difference between PHEV- and mock-inoculated ALI-PRECs (data not shown).

### Increased levels of IL-8 in the subnatants of ALI-PRECs in response to PHEV infection.

**FIG 6:**
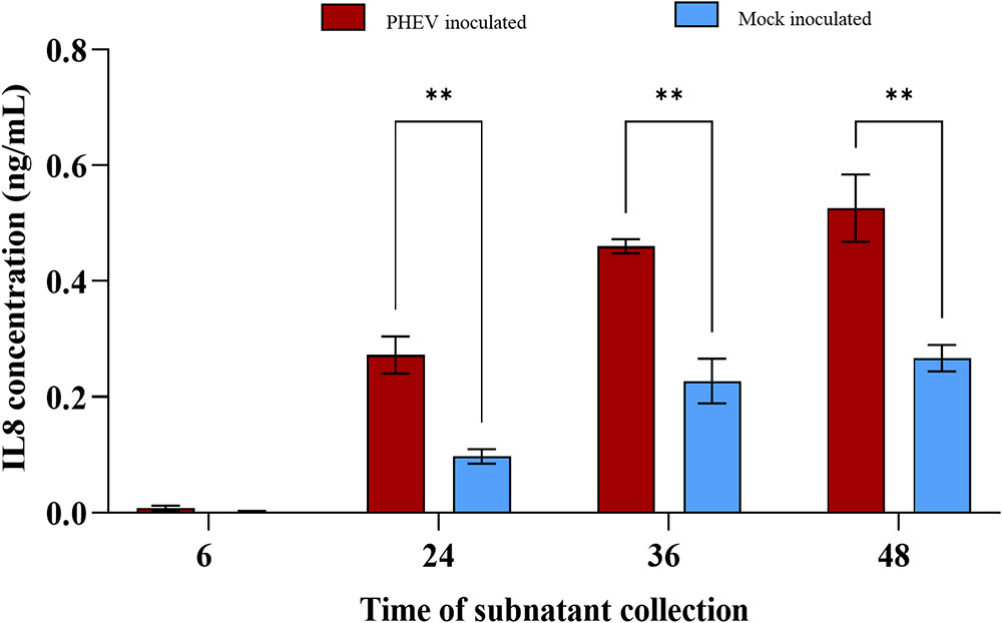
IL-8 secretion into the subnatants of ALI-PRECs treated with PHEV. Subnatants from ALI-PRECs inoculated with PHEV (HA titer of 128) or mock inoculated were collected at 6, 24, 36, and 48 hpi, and the secretory levels of IL-8 were estimated using the Milliplex MAP porcine cytokine/chemokine magnetic bead panel (PCYTMG-23K-13PX; Millipore-Sigma). The minimum detectable concentration (MinDC) of IL-8 was 0.005 ng/ml. This graph was generated from three technical replicates and two biological replicates (2 pigs). Statistical analysis was performed using Fisher’s LSD multiple-comparison test (GraphPad Prism 9.0.1). **, *P* value < 0.005.

After inoculating ALI-PRECs with PHEV and mock medium, the bottom subnatants in the plate well were collected at 6, 24, 36, and 48 hpi and were tested for IFN-γ, IL-1α, IL-1β, IL1Ra, IL-2, IL-4, IL-6, IL-8, IL-10, IL-12, IL-18, tumor necrosis factor alpha (TNF-α), and granulocyte-macrophage colony-stimulating factor (GM-CSF) using a 13-plex Luminex assay. Out of 13 analytes, the minimum detectable concentrations (MinDC; ng/ml) of 12 (gamma interferon [IFN-γ], IL-1α, IL-1β, IL-1Ra, IL-2, IL-4, IL-6, IL-10, IL-12, IL-18, TNF-α, and GM-CSF) analytes were below the range of detection. However, the levels of IL-8 (MinDC, 0.005) increased over time, being significantly (*P* value < 0.005) higher in ALI-PRECs inoculated with PHEV than in mock-inoculated ALI-PRECs (Fig. 6).

## DISCUSSION

### ALI-PRECs are morphologically and functionally similar to the natural epithelial lining of the tracheal region of the porcine respiratory tract.

Together with other major upper respiratory tract organs, the tracheal epithelium constitutes a barrier between the external environment and the lung. However, it does not just provide a mechanical barrier but also primes the initiation, persistence, and resolution of airway mucosal innate immune responses (28). Although ALI-PREC cultures were first reported in 2000 (71), early attempts were unable to determine multilayered differentiated columnar epithelium containing goblet cells. The ALI-PREC culture system developed and characterized in this study exhibited enough complexity to mimic the morphology and physiology of the pseudostratified airway epithelial lining of the tracheal region of the porcine respiratory tract. Ultrastructural analysis by transmitted electron microscopy on differentiated ALI-PRECs (∼27 days of culture) showed a complex pseudostratified columnar epithelium constituted by ciliated and nonciliated airway epithelial cells (i.e., pluripotent proliferative basal cells, M cells, ciliated cells, and mucus-secreting goblet cells), tight junctions, and desmosomes between them (30, 31). Thereby, the ALI-PRECs circumvent the need to use biocontainment animal facilities, reducing the reliance upon the use of tissues from multiple donor animals, or to establish large stocks of primary cells for individual and highly reproducible sets of experiments.

Under ALI conditions, epithelial cells were endowed with beating cilia that, together with the protective mucus layer created by goblet cells, and an airway surface liquid layer (Fig. 2G and N; see also Movie S1 in the supplemental material), mimic *in vivo* the mucociliary clearance apparatus, which is an essential partner of the mucosal immune system (32–36). The epithelial surfaces of ALI-PRECs also expressed different host viral receptors such as sialic acids (37), ACE2 (38), CEACAM1 (39), DPP4 (40), and APN (41), widely used by influenza viruses and coronaviruses. Consequently, ALI cell cultures support viral infections, and they have been used to study host-virus interactions (6, 19–23).

In a previous study, we demonstrated that PHEV (67N strain) infects and replicates in ALI-PRECs derived from the tracheal region of the pig respiratory tract (6). However, the specific host receptor involved in PHEV cell binding and entry and essential steps in establishing viral infections is yet to be determined. Microscopic observations from this and our previous study (6) showed that PHEV causes cytopathic changes and disruption of the ciliated columnar epithelium, i.e., a decrease in the number of cilium-expressing cells and a dampening effect on ciliary movement from 6 to 48 hpi (Movies S1 and S2).

### ALI-PRECs are excellent *ex vivo* models to study molecular immune responses toward PHEV infection.

Previous studies reported the utility of ALI-PREC cultures as infection model for other viruses including porcine respiratory coronavirus (42) and influenza A virus (20, 43). However, to our knowledge, this is the first study to report comparative innate immune responses toward betacoronavirus PHEV *in vivo* and *in vitro*. The current study aimed to investigate the differential gene modulation of PRRs and downstream innate immunity mediators in the upper respiratory tract (i.e., trachea) in response to PHEV infection both *in vivo* (trachea sections from pigs) and *in vitro* (ALI-PREC cultures).

Like other innate immune cells, epithelial cells distinguish pathogens by using an array of PRRs such as TLRs, RLRs, and NLRs (44, 45). Thus, the modulation of key signaling mediators of the innate immunity was evaluated in ALI-PREC cultures inoculated with PHEV 67N over the course of 48 h. Regardless, the same innate immune mediators were also evaluated in tracheal tissue sections collected at necropsy (5 dpi) from 10-day-old CDCD pigs inoculated with the same strain and dose used for ALI-PRECs.

The array of gene expression data presented here shows a consistent upregulation of *TLR3* and/or *TLR7* genes *in vivo* (trachea sections from *in vivo*) and *in vitro* (ALI-PREC cultures) in response to infection, indicating that PHEV RNA might be recognized by these two key PRR upstream innate immune mediators. TLR7 and TLR3 function as an innate host sensor of viral ssRNA and double-stranded RNA (dsRNA), respectively (46), while MyD88 and TRIF are the major adapters that bind to the intracellular domain of TLR to activate the proinflammatory response (47). MyD88 is the canonical adapter for inflammatory signaling pathways downstream of all TLRs except TLR3, which directly recruits the adapter TRIF (47). Generally, MyD88 and TRIF cause nuclear translocation of the transcriptional factor NF-κB, basic leucine zipper family members, and members of the interferon regulatory factor family (IRF3 and IRF7) that ultimately leads to the production of proinflammatory cytokines (e.g., IL-1, IL-6, and TNF-α), which are essential for the antiviral response (48, 49). In the present study, MyD88 but not TRIF was upregulated in response to PHEV infection in ALI-PRECs. Moreover, the gene expression levels of cytoplasmic RLRs RIG-I, recognizing RNA with 5′ triphosphates, and MDA5, which recognizes long and stable dsRNA (50), were upregulated only in ALI-PREC cultures infected with PHEV but not in mock-inoculated control cultures.

Together, the enhanced mRNA levels of *TLR7*, *RIG1*, and *MyD88* signal transduction pathways induced the transcriptional upregulation of type I *IFNα1/β1* genes only in pig tracheal epithelia and of *IFNλ1* in both the tracheal epithelia and ALI-PRECs. These findings in ALI-PRECs are further supported by undetectable levels of secretory levels of IFN-α in the subnatants. While most nucleated cells respond to type I IFN, it has been reported that type III IFN-λ induces IFN-stimulated gene (ISG) expression and efficiently protects from a viral infection of epithelial cells at the intestinal and respiratory tract *in vivo* (51, 52). IFNs induce an autocrine signaling cascade that triggers phosphorylation of signal transducer and activator of transcription (STATs) (53). In this study, the expression of the STAT1 gene was consistently upregulated in both the tracheal epithelia and ALI-PREC cultures. This transcription factor induces the expression of ISGs involved in resistance to viral infections (54, 55). Specifically, we detected significant expression levels of the ISG *OAS1*, which forms part of the OAS/RNase L system (56–58). Like its homologous OAS2 and OAS3, the OAS1 gene encodes an oligoadenylate synthetase that produces short-chain poly(A)s that activate an RNase L, leading to viral dsRNA degradation, and activate other antiviral cell mechanisms beyond the scope of the present study (59). Similarly, an increased expression of the ISG class *Mx1* was observed and identified initially as factors of resistance to lethal influenza A virus infections in mice (60).

Of note, mRNA levels of neither NF-κB (*NF p105*, *NF p50*, and *NFkB2*) nor any of the major proinflammatory cytokines (*IL1β*, *IL6*, and *TNFα*) were differentially regulated in response to PHEV infection. Likewise, a recent study showed that PHEV hampers IFN-β production via inactivation of IRF3 in mouse macrophage cell lines (61), which could suggest an immune evasion tactic of PHEV to subside proinflammatory and type I IFN signaling.

In addition, chemokines are critical for epithelial homeostasis and responses to infections (62, 63). Several chemokine promoters regulating, e.g., CCL2, CCL5, and CXCL10, contain binding sites for STAT1 and/or ISGF3, and their stimulation by type III IFNs enhances secretion of these chemokines. Interestingly, a significant upregulation of *CCL2* and *CCL5* genes in ALI-PRECs, and of *CXCL8* and *CXCL10* genes in both ALI-PRECs and pig trachea sections, was detected in response to PHEV infection. Specifically, the upregulation of *IFNλ1* and *STAT1* genes might play a role in the expression of the chemokines observed in this study. It is well known that the activation of chemokines during infection mediates immune cell trafficking (cellular defense) to the site of infection, and it is a classic response in the respiratory epithelium, having a critical impact on pathogenesis and virus replication and clearance (64–67). In the present study, it was particularly interesting that the upregulation of the *CXCL8/IL8* gene accompanied an increase in the secretory levels of IL-8 in subnatants of ALI-PREC cultures infected with PHEV. This is in line with our previous studies reporting increasing systemic levels of IL-8 in pigs experimentally inoculated with PHEV 67N, leading to the recruitment and subsequent infiltration of T lymphocytes and macrophages (6, 68). Although beyond the scope of this paper, the link between local innate and adaptive immunity will be further investigated using cocultures of ALI-PRECs and immune cells.

### Conclusion.

In summary, the results from this study shed light on the molecular mechanisms driving the innate immune response of the airway epithelium against PHEV and underscore the important role of respiratory epithelial cells in the maintenance of respiratory homeostasis and on the initiation, resolution, and outcome of the infectious process. The ALI-PREC culture established in this study constitutes an excellent *ex vivo* infection model alternative to animal studies, which can be easily extrapolated to different animal species and pathogens.

## MATERIALS AND METHODS

### Porcine hemagglutinating encephalomyelitis virus *in vitro* culture and propagation.

PHEV 67N, or “Mengeling strain,” obtained from the National Veterinary Services Laboratories (NVSL; United States Department of Agriculture [USDA], Ames, IA, USA) was propagated in swine kidney primary (SKP) cells (NVSL) as previously described (10). In brief, SKP cells were maintained in growth medium 1 (GM 1) (minimum essential medium with Earle’s medium [EMEM] [Thermo Fisher Scientific Inc., Waltham, MA, USA] with 0.5% lactalbumin enzymatic hydrolysate [Millipore-Sigma, Burlington, MA, USA] supplemented with heat-inactivated 10% fetal bovine serum [FBS; ATCC, Manassas, VA, USA], 0.15% sodium bicarbonate [Millipore-Sigma], 1% l-glutamine [Thermo Fisher Scientific], 1% sodium pyruvate [Thermo Fisher Scientific], 3 μg/ml amphotericin B [AmpB; Thermo Fisher Scientific], 25 μg/ml kanamycin [Thermo Fisher Scientific], and 75 μg/ml of gentamicin [Thermo Fisher Scientific]), at 37°C with 5% CO_2_. Cells were inoculated with 2 ml of antibiotic-trypsin-Versene (ATV) trypsin once 80% confluence was achieved in a 75-cm^2^ tissue culture flask (Thermo Fisher Scientific) and incubated for 5 min at 37°C with 5% CO_2_. Subsequently, 5 ml of virus diluted 1:10 in infection medium for SKP cells (GM 1 without FBS) was added and incubated for 4 days at 37°C with 5% CO_2_. The virus was harvested and titrated by hemagglutination assay and stored at −80°C.

### Experimental inoculation of CDCD neonatal pigs.

The experimental protocol for the animal study was approved by the Institutional Animal Care and Use Committee (IACUC log no. 12-17-8658-S; approval date, 3 January 2018) of Iowa State University (ISU). The animal study was fully described in a previous study (10). Briefly, 7-day-old CDCD piglets (Yorkshire × Large White crossbred) were inoculated with 5 ml of virus inoculum (0.5 ml of PHEV 67N [1:128 hemagglutinin {HA}] in 4.5 ml of EMEM [Thermo Fisher Scientific]) oronasally or mock inoculated (control group) with 5 ml of EMEM oronasally.

### Tissue collection.

Tissues from the tracheal region of the respiratory tract, i.e., from below the larynx to the bronchial bifurcation (approximately 6 to 8 in.), were aseptically collected from piglets immediately after necropsy. Some samples from both PHEV-inoculated and control animals were dissected and fixed in 10% buffered neutral formalin or snap-frozen in liquid nitrogen and stored at −80°C for gene expression analysis. In addition, tracheal sections of healthy piglets within the negative-control group were collected in Dulbecco’s minimum essential/Ham’s F-12 medium with GlutaMAX (DMEM/F-12) (Thermo Fisher Scientific), supplemented with 100 IU/ml of penicillin/100 μg/ml of streptomycin (Pen-Strep) (Thermo Fisher Scientific) and 1.25 μg/ml of amphotericin B (AmpB) (Thermo Fisher Scientific) for isolation and subculture of PRECs.

### Culture of primary porcine respiratory epithelial cells at an air-liquid interface.

Isolation and culture of PRECs were performed as previously described (6, 21). Tracheal samples were washed and incubated in phosphate-buffered saline (PBS) supplemented with Pen-Strep to remove any blood clots. Then, samples were incubated at 4°C for 48 h in digestion medium (calcium- and magnesium-free minimum essential medium [MEM; in-house]), supplemented with 1.4 mg/ml pronase (Millipore-Sigma), 0.1 mg/ml DNase (Millipore-Sigma), and 100 μg/ml Primocin (InvivoGen, San Diego, CA, USA). Tissue digestion was neutralized using 10% heat-inactivated EqualFetal FBS (Atlas Biologicals, Fort Collins, CO, USA). The tissue digest containing cells was passed through a 40-μm cell strainer, washed, pelleted, and resuspended in DMEM/F-12. The cells collected were either seeded directly using respective growth medium or frozen in LHC basal medium (Thermo Fisher Scientific) containing 30% FBS and 10% dimethyl sulfoxide (DMSO) (Millipore-Sigma). These primary cells were not subjected to further subculturing.

Isolated PRECs were seeded at a density of ∼20,000 cells/mm^2^ on 24-well ThinCert cell culture inserts or “transwell inserts” (Greiner Bio-One North America Inc., Monroe, NC, USA) previously coated with collagen from the human placenta—Bornstein and Traub type IV (Millipore-Sigma). For the first 24 h, cells were grown in growth medium 2 (GM 2) containing DMEM/F-12 supplemented with 10% FBS, 1× MEM nonessential amino acids, Pen-Strep, and AmpB at 37°C and 5% CO_2_. After 24 h, GM 2 was removed from plate well and transwell inserts. Subsequently, growth medium 3 (GM 3) containing DMEM/F-12 supplemented with 1,400 nM hydrocortisone (Acros Organics, Fair Lawn, NJ, USA), 2,700 nM epinephrine (Acros Organics), 100 nM retinoic acid (Acros Organics), 9.7 nM 3,3′,5-triiodo-l-thyronine (Cayman Chemicals, Ann Arbor, MI, USA), 0.5 ng/ml murine epidermal growth factor (EGF) (PeproTech US, Rocky Hill, NJ, USA), 1× insulin-selenium-transferrin (Thermo Fisher Scientific), 1× HEPES (Thermo Fisher Scientific), 2% Ultroser-G (Pall France, Cergy, France), Pen-Strep, and AmpB was used to replace only medium in the plate well. GM 3 was replaced every 2 to 3 days until PRECs were completely differentiated into ALI-PRECs. On day 18 postseeding, PRECs on transwells were completely confluent with no visible medium seepage, and a shiny glaze that resembled mucus was noticed on top of all the cultures. Cilium cell development and differentiation continued through days 27 to 30. Upon complete differentiation, ALI-PRECs were then used for PHEV infection studies or further characterization. Micrographs were captured using relief contrast settings of an Olympus CKX4 microscope (Olympus Corp., Center Valley, PA, USA), an Infinity 2 camera, and Infinity Analyze imaging software (ver. 6.5.5; Lumenera Corp., Ottawa, ON, Canada).

### Cellular characterization of ALI-PRECs.

Differentiated and confluent ALI-PRECs on transwell inserts were fixed with 1:1 acetone-methanol (intracellular staining) or 4% paraformaldehyde and carefully embedded in paraffin. Cross sections of paraffin-embedded ALI-PRECs on transwell inserts were used for various staining procedures described here. Micrographs were captured using relief contrast settings of an Olympus CKX4 microscope, an Infinity 2 camera, and Infinity Analyze imaging software (version 6.5.5; Lumenera Corp., Ottawa, ON, Canada).

### Mucin staining.

Neutral and acidic mucins on ALI-PRECs were analyzed using an alcian blue and periodic acid-Schiff (PAS) stain. For alcian blue staining, deparaffinized and hydrated ALI-PREC sections were treated with 3% acetic acid for 3 min, followed by incubation with alcian blue (pH 2.5) for 30 min. The sections were counterstained with nuclear fast red for 5 min. For PAS staining, sections were treated with 1% periodic acid for 10 min, followed by incubation with Schiff’s reagent for 15 min. The sections were counterstained with fast green for 1 min. Following staining, dehydrated sections were cleared in Histo-Clear II (Electron Microscopy Sciences [EMS], Hatfield, PA, USA) and mounted in Tissue-Tek Glas mounting medium (Sakura Finetek U.S.A., Inc., Torrance, CA, USA).

### Lectin cytochemistry.

ALI-PRECs were characterized for sialic acids using a lectin cytochemistry assay previously described (37). In brief, cross sections of ALI-PRECs on transwell inserts and fixed with 4% paraformaldehyde were presoaked in Tris-buffered saline (TBS) and blocked using a biotin-streptavidin blocking kit (Vector Laboratories Inc., Burlingame, CA, USA) according to manufacturer’s instructions. The sections were incubated overnight at 4°C with Sambucus nigra lectin (SNA), which binds preferentially to α-2,6-linked sialic acids, and Maackia amurensis II lectin (MAL II), binding to α-2,3-linked sialic acids, both at a final concentration of 10 μg/ml. Subsequently, the cells were incubated with 2 μg/ml streptavidin-Dy650 conjugate (Thermo Fisher Scientific) for 2 h at room temperature and counterstained with NucBlue fixed-cell ReadyProbes reagent (Thermo Fisher Scientific).

### Immunocytochemistry.

Immunocytochemistry was performed for the detection of various cell markers of interest using commercially available antibodies {i.e., tight junction marker occludin [sc-133256; Santa Cruz Biotechnology, Dallas, TX, USA], cell proliferation marker Ki67 [sc-23900; Santa Cruz Biotechnology], human ACE2 [amino acids 631 to 805; sc-390851; Santa Cruz Biotechnology], human CEACAM1/CD66a [amino acids 391 to 526; sc-166453; Santa Cruz Biotechnology], DPP4/CD26 [CACT114A-BOV2078; The Washington State University Monoclonal Antibody Center, Pullman, WA, USA], human APN/CD13 receptors [amino acids 668 to 967; sc-166105; Santa Cruz Biotechnology], epithelial cell marker pancytokeratin [MCA1907T; Bio-Rad Laboratories, Hercules, CA, USA], microfold [M] antibody [clone NKM 16-2-4 specifically binds (1,2)-fucose-containing carbohydrate moieties; 130-096-148; Miltenyi Biotec Inc., Auburn, CA, USA]}. In this study, the ImmPRESS VR anti-mouse IgG horseradish peroxidase (HRP) polymer detection kit (MP-6402-15; Vector Laboratories) was used as per the manufacturer’s instructions. Briefly, ALI-PREC sections were incubated overnight at 4°C with the respective primary mouse monoclonal antibodies followed by 2 h of incubation with the following secondary antibodies. A horse-raised anti-mouse IgG polymer labeled with horseradish peroxidase (HRP) (Vector Laboratories) was used for chromogenic stains. Next, cells were incubated in 3,3-diaminobenzidine (DAB) HRP substrate for 5 min and counterstained with hematoxylin and eosin (H&E). For fluorogenic detection, a goat-raised anti-mouse secondary antibody labeled with Alexa 647 (Jackson ImmunoResearch, West Grove, PA, USA) was used and counterstained with NucBlue fixed cell ReadyProbes reagent (Thermo Fisher Scientific).

### Transmitted electron microscopy.

Transwell membranes carrying differentiated ALI-PRECs were fixed with 1% paraformaldehyde and 3% glutaraldehyde in 0.1 M sodium cacodylate buffer, pH 7.2, for 48 h at 4°C and then cut into small 2-mm^2^ squares. Samples were washed in cacodylate buffer (Electron Microscopy Sciences, Hatfield, PA, USA) three times (10 min each) and postfixed with 1% osmium tetroxide in 0.1 M sodium cacodylate buffer for 1 h at room temperature. Samples were washed with deionized water three times (15 min each) and *en bloc* stained using 2% uranyl acetate in distilled water for 1 h. Samples were washed in distilled water for 10 min and dehydrated through a graded ethanol series (25, 50, 70, 85, 95, and 100%) for 1 h for each step. Samples were further dehydrated with three changes of pure acetone (15 min each) and infiltrated with EmBed 812 formula (hard) for Epon epoxy resin (Electron Microscopy Sciences) with graded ratios of resin to acetone until thoroughly infiltrated with pure epoxy resin (3:1, 1:1, 1:3, and pure) for 6 to 12 h per step. Samples were placed into Beem capsule lids and were polymerized at 70°C for 48 h. Thick sections (1.5 μm) were made using a Leica UC6 ultramicrotome (Leica Microsystems, Buffalo Grove, IL, USA) and stained with EMS epoxy stain (a blend of toluidine blue-O and basic fuchsin) to identify areas of interest for transmitted electron microscopy (TEM). Thin sections were made at 50 nm and collected onto single-slot carbon film grids. TEM images were collected using a 200-kV JEOL JSM 2100 scanning transmission electron microscope (Japan Electron Optics Laboratories, Peabody, MA, USA) with a Gatan One View 4K camera (Gatan Inc., Pleasanton, CA, USA).

### PHEV infection of ALI-PREC cultures.

Completely differentiated ALI-PREC cultures were inoculated with 250 μl of a 1:1 dilution of PHEV 67N (1:128 HA titer) and infection medium for ALI-PRECs containing DMEM/F-12 supplemented with 2% Ultroser G, 1× MEM nonessential amino acids, 1× HEPES, Pen-Strep, and 2 μg/ml *N*-tosyl-l-phenylalanine chloromethyl ketone (TPCK)-treated trypsin (Millipore-Sigma) or mock inoculated with infection medium and incubated for 6 h at 37°C and 5% CO_2_. Then, the inoculum was removed, the cell cultures were washed once with DMEM/F-12, fresh infection medium was added into the plate wells, and plates were incubated for 24, 36, and 48 h postinoculation (hpi) at 37°C with 5% CO_2_. ALI-PREC cultures were monitored daily under the microscope for the presence of cytopathic changes, and subnatants were collected at different time points over the course of the infection for assessment of virus replication.

### PHEV RT-qPCR.

Viral RNA extractions were performed using the E.Z.N.A. viral RNA kit (Omega Bio-tek, Inc., Norcross, GA, USA) and vacuum manifold (Qiagen, Germantown, MD, USA) method following the manufacturer’s instructions. A quantitative PHEV N-gene-based RT-PCR (RT-qPCR) developed by Tetracore (Tetracore, Inc., Rockville, MD, USA) and ISU (68) was used to confirm and quantify PHEV infection *in vivo* (CDCD pigs) and *ex vivo* (ALI-PREC cultures). Each RT-qPCR mixture (25-μl final reaction volume) was set up by combining 19 μl of PHEV RT-qPCR master mix and 1 μl of the enzyme blend (reverse transcriptase and RNase inhibitor). An internal control (IC) was used as an extraction control, with 6 μl of the IC added to the lysis buffer. Then, 5 μl of the extracted sample RNA with IC was added to Master Mix. All RT-qPCRs were performed in duplicate, with a negative extraction control (NEC), positive extraction control (PEC), and a “no-template” control (NTC) included in each run. RT-qPCRs were run on a Rotor-Gene Q (Qiagen) under cycling conditions of 48°C for 15 min and 95°C for 2 min for holding and 45 cycles of 95°C for 10 s for denaturation and 60°C for 40 s for amplification. The RT-qPCR results were analyzed using Rotor-Gene Q Pure Detection software (v 2.3.1). Samples with a threshold cycle (*C_T_*) above 40 were considered negative.

### Total RNA isolation, reverse transcription, and transcriptional analysis.

For immunological characterization, total RNA was isolated from cells or snap-frozen tissues. Cells were lysed with TRIzol reagent (Thermo Fisher Scientific), while snap-frozen swine tissues were homogenized in TRIzol reagent using 2.8-mm ceramic beads (Omni). After performing the chloroform phase separation, total RNA was extracted from all samples using a commercially available RNeasy Plus minikit (Qiagen), according to the manufacturer’s protocol. Eluted RNA was quantified using a NanoDrop One Microvolume UV-visible (UV-Vis) spectrophotometer (Thermo Fisher Scientific). Samples with an *A*_260/280_ between 1.96 and 2.05 were used for reverse transcription using the qScript XLT cDNA SuperMix kit (Quantabio, Beverly, MA, USA).

This study involved transcriptional analysis of 45 target genes (see Table S1 in the supplemental material), including seven endogenous controls (*ACTB*, *B2M*, *EIF3K*, *GAPDH*, *PPIA*, *RPL10*, and *PCNA*) in both ALI-PRECs and trachea from experimentally infected pigs. The genes related to PRRs and their downstream mediators, antigen-presenting molecules, transcriptional regulators, mediators of inflammation and cell death, classical antiviral genes, interferon stimulatory genes, proinflammatory cytokines, and chemokines were analyzed.

10.1128/mSphere.00820-21.1 TABLE S1 List of primers used in this study. Download Table S1, PDF file, 0.3 MB. Copyright © 2021 Nelli et al. 2021 Nelli et al. https://creativecommons.org/licenses/by/4.0/ This content is distributed under the terms of the Creative Commons Attribution 4.0 International license.

All qPCRs were performed using 1× PowerUp SYBR green master mix (Thermo Fisher Scientific), 500 nM swine-specific primers (Table S1; Millipore-Sigma), and 1.5 ng (cells) and 20 ng (tissues) of total RNA converted to cDNA. For 96-well format, the Applied Biosystems 7500 Fast real-time system (Applied Biosystems, Foster City, CA, USA), and for high-throughput format, the Wafergen SmartChip real-time PCR system (TaKaRa Bio USA, Inc., Mountain View, CA, USA) were used. Each run of the SmartChip real-time PCR system can perform 5,184 real-time PCRs with a volume of 100 ml each, and they were filled using the SmartChip multisample nanodispenser. Quality assurance/quality control checks were performed according to a standard protocol provided by Wafergen Biosystems (service provider RTSF, Michigan State University, East Lansing, MI, USA). The cycling conditions used were 50°C for 2 min and 95°C for 2 min of holding and 40 cycles of 95°C for 15 s for denaturation and 60°C for 1 min for amplification; final melting curve analysis was performed at 95°C for 15 s, 60°C for 1 min, and 95°C for 15 s. All qPCRs were performed in duplicate, and no-template controls (NTCs) were included in each plate/chip.

Finally, qPCR results were analyzed either using 7500 software v2.3 (Applied Biosystems) or SmartChip qPCR software v 2.8.6.1 (TaKaRa Bio USA, Inc.) and exported to Microsoft Excel. Amplification efficiencies beyond the range (1.5 to 2.2) and samples with multiple melting peaks and above a *C_T_* value of 40 were discarded. *C_T_* values were subsequently analyzed on qBase+ gene expression analysis software (Bio Gazelle, Zwijnaarde, Belgium), which calculates the stability of endogenous control genes and provides a value called M-value. Out of seven endogenous control genes in both PHEV-treated and mock-treated cells, three genes (*EIF3K*, *PPIA*, and *RPL10*) were identified as the best endogenous controls based on their low M-value. Hence, gene expression data were normalized using the geometric mean of these three genes (69). Relative quantitation analysis was performed using the ΔΔ*C_T_* method as described previously (70).

### Multiplex porcine cytokine and chemokine immunoassay.

Using the bottom (plate well) subnatants collected from ALI-PRECs inoculated with PHEV or culture medium (negative control), at 6, 24, 36, and 48 hpi, a porcine cytokine and chemokine 13-plex Luminex assay, including IFN-γ, IL-1α, IL-1β, IL-1Ra, IL-2, IL-4, IL-6, IL-8, IL-10, IL-12, IL-18, TNF-α, and GM-CSF (Milliplex MAP porcine cytokine/chemokine magnetic bead panel; Millipore-Sigma), was performed following manufacturer’s instructions (i.e., using overnight protocol). The 96-well plates were analyzed using a Bio-Plex 200 system operated by the Bio-Plex Manager software (Bio-Rad, Hercules, CA, USA). The mean fluorescence intensity data of each sample were subtracted from the blank wells, and the concentration of each cytokine was calculated from the standard curve (5-parametric logistic) generated from the kits’ internal standards and analyzed using GraphPad Prism 8 (GraphPad Software Inc., La Jolla, CA, USA).

### Data analysis.

Tracheal tissue sections were collected at necropsy (5 dpi) from two CDCD neonatal pigs in the mock-inoculated control group and two piglets in the PHEV-inoculated group. Meanwhile, the primary epithelial respiratory cells used for the development of ALI-PREC cultures were obtained from two independent control CDCD pigs, with two or three technical culture replicates used for infection or qPCR assays, respectively. Statistical analyses and plots were performed using GraphPad Prism 9.0.2 (GraphPad Software Inc.). The statistical significance was determined using the two-way analysis of variance (ANOVA) multiple comparisons of Fisher’s least significant difference (LSD) test. For all analyses, a *P* value of <0.05 was considered statistically significant.
