# Supplementary material for: The Betacoronavirus PHEV Replicates and Disrupts the Respiratory Epithelia and Upregulates Key Pattern Recognition Receptor Genes and Downstream Mediators, Including IL-8 and IFN-λ
Source: mSphere. 2021 Dec 22;6(6):e00820-21. doi: 10.1128/mSphere.00820-21 (PMC8694173; doi:10.1128/mSphere.00820-21)
Supplement: TABLE S1 [file msphere.00820-21-st001.pdf]

Supplemental Table S1. List of primers used in this study.

| Gene | NCBI accession | Forward Primer (5'-3') | Reverse Primer (5'-3') | Amplicon size | Ref |
|---|---|---|---|---|---|
| *ACTB* | AY550069.1 | CTCTTCCAGCCCTCCTTCCT | CGACGTCGCACTTCATGATG | 82 | # |
| *B2M* | NM_213978.1 | CCCCCGAAGGTTCAGGTTTA | GCAGTTCAGGTAATTTGGCTTTC | 66 | # |
| *EIF3K* | XM_005664595.3 | ACCAGTTCAACCCAGCTTTCTTC | GGTTGGTGAGGGCCTTCAG | 69 | # |
| *GAPDH* | NM_001206359.1 | CCCCAACGTGTCGGTTGT | CCTGCTTCACCACCTTCTTGA | 83 | # |
| *PPIA* | NM_214353.1 | GGTCCTGGCATCTTGTCCAT | TGGCAGTGCAAATGAAAAACTG | 73 | # |
| *RPL10* | NM_001044543.1 | CCACATTGGCCAAGTCATCAT | CTGCGCAGGGCCTCAA | 75 | # |
| *PCNA* | NM_001291925.1 | TACGCTAAGGGCAGAAGATAATGC | TGAGATCTCGGCATATACGTG | | # |
| *TLR3* | NM_001097444.1 | GCGGTCCTGTTCAGTTTCT | AAGGCATCTGCTGGGATTT | 91 | (1) |
| *TLR7* | NM_001097434.1 | CCCAGGTCCTCGAATCATTAC | CATTAAGAGGCAAGGAGGAAGA | 99 | (1) |
| *TLR9* | NM_213958.1 | CCTCACACATCTCTCACTCAAG | GGTGACAATGTGGTTGTAGGA | 103 | (1) |
| *TNFα* | NM_214022.1 | CCTACTGCACTTCGAGGTTATC | GCATACCCACTCTGCCATT | 177 | (1) |
| *IL1β* | NM_214055.1 | ACCTGGACCTTGGTTCTCTG | CATCTGCCTGATGCTCTTGT | 83 | (2) |
| *IL6* | NM_214399.1 | CAAGGAGGTACTGGCAGAAA | CAGCCTCGACATTTCCCTTAT | 185 | (1) |
| *IL8/CXCL8* | NM_213867.1 | CCGTGTCAACATGACTTCCAA | GCCTCACAGAGAGCTGCAGAA | 75 | (3) |
| *IL12A/IL12p35* | NM_213993.1 | CAGGCCCAGGAATGTTCAAA | CGTGGCTAGTTCAAGTGGTAAG | 188 | (1) |
| *CXCL10* | NM_001008691.1 | CTGCATCAAGATCAGTGACAGAC | TTGTGGCAATGATCTCAACAT | 104 | (2) |
| *MCP1/CCL2* | NM_214214.1 | GCAGCAAGTGTCCTAAAGAAGCA | GCTTGGGTTCTGCACAGATCT | 72 | (4) |
| *RANTES/CCL5* | NM_001129946.1 | AGCATCAGCCTCCCCATATG | TTGCTGCTGGTGTAGAAATATTCC | 108 | (4) |
| *RIG-I/DDX58* | NM_213804.2 | GAGCCCTTGTGGATGCTTTA | GGGTCATCCCTATGTTCTGATTC | 114 | (1) |
| *TRIF/TICAM1* | MK302497.1 | CTCCGGTGCAGTCAAACA | GGTAGTGTGTGCTGGTTTCT | 111 | (1) |
| *TRAF3* | XM_021081628.1 | CTCCTCCAGCCCAAAATGTA | TTCTTCAAATGCACCAGCAG | 171 | (5) |
| *TRAF6* | NM_001105286.1 | GGGAACGATACGCCTTACAA | CTCTGTCTTAGGGCGTCC | 174 | (1) |
| *MyD88* | NM_001099923.1 | GGCAGCTGGAACAGACCAA | GGCAGGACATCTCGGTCAGA | 61 | (1) |
| *IRF7* | NM_001097428.1 | CACTACACAGAGAAGCTGCT | ACCTCCCAGTAGACTTTGC | 110 | (1) |
| *MDA5/IFIH1* | XM_005671881.3 | CAGTGTGCTAGCCTGCTCTG | GCAGTGCCTTGTTTCCTCTC | 113 | (6) |
| *NFkB1 (p105)* | NM_001048232.1 | GAGGTGCATCTGACGTATTC | CACATCTCCTGTCACTGCAT | 138 | (1) |
| *NFkB1 (p50)* | KC316024.1 | AAGCACGGAACTGTAGACAC | TCTGTGGTTTCTGTGACTTTCC | 107 | (1) |
| *NFkB2* | XM_021072741.1 | GACACCTTGGCGTTGTTAACC | CCCGTGATGACTGCCAAGT | 70 | # |
| *IFNα1* | M28623.1 | GCCTCCTGCACCAGTTCTACA | TGCATGACACAGGCTTCCA | 57 | (7) |
| *IFNβ1* | M86762 | AGTGCATCCTCCAAATCGCT | GCTCATGGAAAGAGCTGTGGT | 59 | (8) |
| *IFNγ* | NM_213948.1 | CCATTCAAAGGAGCATGGAT | TTCAGTTTCCCAGAGCTACCA | 76 | (6) |
| *IFNλ1/IL-29* | NM_001142837.1 | GGTGCTGGCGACTGTGATG | GATTGGAACTGGCCCATGTG | 102 | (9) |
| *OAS1* | NM_214303.2 | GAGCTGCAGCGAGACTTCCT | TGCTTGACAAGGCGGATGA | 68 | (4) |
| *Mx1* | NM_214061.2 | GAGGTGGACCCCGAAGGA | CACCAGATCCGGCTTCGT | 57 | (8) |
| *EGFR* | NM_214007.1 | GGCCTCCATGCTTTTGAGAA | GACGCTATGTCCAGGCCAA | 95 | (10) |
| *MHC1* | NM_001097431.1 | TGCGCGGCTACTACAACCA | TCCCAAGTAGCAGCCAAACAT | 71 | # |
| *MHC2* | NM_001130224 | TTGCCTCCTATGGCTTAAATGTC | TCGTCGCCATCAAATTCATG | 76 | # |
| *NLRP3* | NM_001256770.2 | CCTCTTTGGCCTTGTAAACC | TGGCTGGGCTCAATCTGTAG | 144 | (11) |
| *NOD1* | NM_001114277.1 | CTGTCGTCAACACCGATCCA | CCAGTTGGTGACGCAGCTT | 57 | (12) |
| *NOD2* | NM_001105295.1 | GAGCGCATCCTCTTAACTTTCG | ACGCTCGTGATCCGTGAAC | 66 | (12) |

| | | | | | |
|---|---|---|---|---|---|
| *IRF3* | NM_213770.1 | GCTACACCCTCTGGTTCTGC | GAGACACATGGGGACAACCT | 95 | (6) |
| *RIPK1* | XM_005665537.3 | GCGAGGGCGTCATCCA | CGGCGATCTTGATGTGAAAGT | 75 | # |
| *AP1* | NM_213880.1 | TGCGGCCCCGAAACT | AGTCATAGAACAGTCCGTCACTTCA | 68 | # |
| *SOCS1* | NM_001204768.1 | TTCTTCGCCCTCAGTGTGAA | GGCCTGGAAGTGCACGC | 63 | (3) |
| *SOCS3* | NM_001123196.1 | CACTCTCCAGCATCTCTGTC | TCGTACTGGTCCAGGAACTC | 105 | (13) |
| *STAT1* | NM_213769.1 | GAAAGTACTACTCCAGGCCAAAGGA | CACAGAAATCAATTCAGTCTTGATGTATC | 95 | (3) |
| *STAT3* | NM_001044580.1 | GCTTTATCAGTAAGGAGA | CAGCGGAGACACAAGGAT | 251 | (14) |
| *STAT4* | NM_001197305.1 | GAAAACCCTCTGAAGTACCTCTATCCT | TCACATGGCTGGGAGCTGTA | 80 | (3) |
| *PCNA* | *NM_001291925.1* | TACGCTAAGGGCAGAAGATAATG | CTGAGATCTCGGCATATACGTG | 191 | (15) |
| *MUCIN2* | XM_021082584.1 | GGCTGCTCATTGAGAGGAGT | ATGTTCCCGAACTCCAAGG | 214 | (16) |
| *CHGA* | NM_001164005.2 | GACCTCGCTCTCCAAGGAGCCA | TGTGCGCCTGGGCGTTTCTT | 332 | (16) |
| *LGR5* | XM_021090898.1 | GGTCGGTTGCCAAATCGTT | TCCAGGGCTGCCAGAGTAAG | 81 | # |
| *SPIB isoform 1* | XM_003127365.3 | GCCAAGACCGGCGAGAT | CAGCGCACTGTCAAACTGGTA | 63 | # |
| *GP2* | XM_003124571.4 | GGTTTCTCTCTGCGAGTTCCTTAA | CTGGGTCGATAGCTCCTCCTT | 89 | # |
| *KRT5* | XM_003126173.4 | AGGTCACCGTCAACCAGAGTCT | GGTCCTCACCCGCTGGAT | 74 | # |
| *DPP4/CD26* | NM_214257.1 | ACCAGGACTCTCAGCCCAAA | ACAAGTAGTGATCCCCTATTAACACAGA | 85 | # |
| *APN/ANPEP* | NM_214277.1 | CACGACACAGATGCAGTCTACAGA | TGTTGAACGTGGCCTTCATG | 80 | # |
| *ACE2* | NM_001123070.1 | GGGTGGTGATGGGATTGGTA | TTGCTTTTTCTTCCTTCGATCTCT | 80 | # |
| *CEACAM1/CD66a* | XM_021094420.1 | TGCTCGCAGAGAGGATAAAACTG | GGCCTCGCACTGATAATTCC | 95 | # |

*# designed for this study*


1. Temeeyasen G, Sinha A, Gimenez-Lirola LG, Zhang JQ, Pineyro PE. 2018. Differential gene modulation of pattern-recognition receptor TLR and RIG-I-like and downstream mediators on intestinal mucosa of pigs infected with PEDV non S-INDEL and PEDV S-INDEL strains. Virology2017/12/19. 517:188–198.

2. Zhang Q, Ma J, Yoo D. 2017. Inhibition of NF-κB activity by the porcine epidemic diarrhea virus nonstructural protein 1 for innate immune evasion. Virology 510:111–126.

3. Royaee AR, Husmann RJ, Dawson HD, Calzada-Nova G, Schnitzlein WM, Zuckermann FA, Lunney JK. 2004. Deciphering the involvement of innate immune factors in the development of the host response to PRRSV vaccination. Veterinary Immunology and Immunopathology 102:199–216.

4. Gudmundsdottir I, Risatti GR. 2009. Infection of porcine alveolar macrophages with recombinant chimeric porcine reproductive and respiratory syndrome virus: Effects on cellular gene transcription and virus growth. Virus Research 145:145–150.

5. Yang Q, Pröll MJ, Salilew-Wondim D, Zhang R, Tesfaye D, Fan H, Cinar MU, Große-Brinkhaus C, Tholen E, Islam MA, Hölker M, Schellander K, Uddin MJ, Neuhoff C. 2016. LPS-induced expression of CD14 in the TRIF pathway is epigenetically regulated by sulforaphane in porcine pulmonary alveolar macrophages. Innate Immunity 22:682–695.

6. Brogaard L, Heegaard PMH, Larsen LE, Mortensen S, Schlegel M, Dürrwald R, Skovgaard K. 2016. Late regulation of immune genes and microRNAs in circulating leukocytes in a pig model of influenza A (H1N2) infection. Scientific Reports 6:1–11.

7. Loving CL, Brockmeier SL, Sacco RE. 2007. Differential type I interferon activation and susceptibility of dendritic cell populations to porcine arterivirus. Immunology 120:217–229.

8. de los Santos T, de Avila Botton S, Weiblen R, Grubman MJ. 2006. The Leader Proteinase of Foot-and-Mouth Disease Virus Inhibits the Induction of Beta Interferon mRNA and Blocks the Host Innate Immune Response. Journal of Virology 80:1906–1914.

9. Wang D, Fang L, Liu L, Zhong H, Chen Q, Luo R, Liu X, Zhang Z, Chen H, Xiao S. 2011. Foot-and-mouth disease virus (FMDV) leader proteinase negatively regulates the porcine interferon-λ1 pathway. Molecular Immunology 49:407–412.

10. Yang L, Xu J, Guo L, Guo T, Zhang L, Feng L, Chen H, Wang Y. 2018. Porcine Epidemic Diarrhea Virus-Induced Epidermal Growth Factor Receptor Activation Impairs the Antiviral Activity of Type I Interferon. Journal of Virology 92.

11. Park H 1980-. 2019. Characterization of Porcine Nlrp3 Inflammasome-Mediated Interleukin-1 Beta Production in Response To Influenza a Viruses.

12. Iida H, Tohno M, Islam MdA, Sato N, Kobayashi H, Albarracin L, Kober AH, Ikeda-Ohtsubo W, Suda Y, Aso H, Nochi T, Miyazaki A, Uenishi H, Iwabuchi N, Xiao J, Villena J, Kitazawa H. 2019. Paraimmunobiotic Bifidobacteria Modulate the Expression Patterns of Peptidoglycan Recognition Proteins in Porcine Intestinal Epitheliocytes and Antigen Presenting Cells. Cells 8:891.

13. Delgado-Ortega M, Melo S, Meurens F. 2011. Expression of SOCS1-7 and CIS mRNA in porcine tissues. Veterinary Immunology and Immunopathology 144:493–498.

14. Hall VJ, Christensen J, Gao Y, Schmidt MH, Hyttel P. 2008. Porcine pluripotency cell signaling develops from the inner cell mass to the epiblast during early development. Developmental Dynamics 238:2014–2024.

15. Willing BP, Van Kessel AG. 2007. Enterocyte proliferation and apoptosis in the caudal small intestine is influenced by the composition of colonizing commensal bacteria in the neonatal gnotobiotic pig. Journal of Animal Science 85:3256–3266.

16. Gonzalez LM, Williamson I, Piedrahita JA, Blikslager AT, Magness ST. 2013. Cell Lineage Identification and Stem Cell Culture in a Porcine Model for the Study of Intestinal Epithelial Regeneration. PLoS One 8:e66465.
